# Theoretical Analysis of Blast Protection of Graded Metal Foam-Cored Sandwich Cylinders/Rings

**DOI:** 10.3390/ma13173903

**Published:** 2020-09-03

**Authors:** Minzu Liang, Xiangyu Li, Yuliang Lin, Kefan Zhang, Fangyun Lu

**Affiliations:** College of Liberal Arts and Sciences, National University of Defense Technology, Changsha 410073, China; xiangyulee@nudt.edu.cn (X.L.); yulianglin@nudt.edu.cn (Y.L.); fly3away@163.com (K.Z.); fylu@nudt.edu.cn (F.L.)

**Keywords:** blast protection, sandwich structure, metallic foams, gradient, cylinders

## Abstract

The blast resistance of a sandwich-walled cylinder/ring comprising two metal face-sheets and a graded metal foam core, subjected to internal air blast loading, is investigated. Analytical models are developed for the deformation of the sandwich cylinder with positive and negative gradient cores under internal blast loading. The deformation process is divided into three distinct phases, namely the fluid–structure interaction phase, core-crushing phase, and outer face-sheet deformation phase. Finite element modeling is performed using the Voronoi material model. The proposed analytical models are verified through finite element analysis, and reasonable agreement is observed between the analytical predictions and finite element results. The sandwich structures with high energy absorption capacity or low maximum radial deflection are satisfied for the protecting purpose of impact/blast resistance requirements. Typical deformation processes are classified and analyzed; the effects of explosive charge, face-sheet thickness, and core gradient on the structural response are also examined. The results indicate that both the deformation modes and the structural response of the cylinders are sensitive to the blast charge and core configuration. It is concluded that energy absorption capacity and maximum radial deflection are two conflicting goals for achieving high impact/blast resistance capability. An in-depth understanding of the behavior in sandwich-walled cylinders under blast impulse and the influence of the core configuration helps realize the advantages and disadvantages of using graded foam materials in sandwich structures and can provide a guideline for structural design.

## 1. Introduction

Sandwich structures generally consist of two metal face-sheets and a foam core [1,2]. In the past three decades, sandwich structures were widely used in aerospace, marine, and other novel impact/blast resistant structures because of their excellent performance of ultra-light, high stiffness and strength to weight ratios, and effective energy absorption (EA) capacity under impact loading [3,4,5]. Dynamic responses of such composite sandwich structures subjected to impact/blast loading have been extensively studied [6,7,8]. Recently, graded cellular materials, in which the mechanical properties vary gradually or layer-by-layer, were always utilized as cores in sandwich composites [9,10,11]. Sandwich structures with graded core configurations have elicited increasing attention recently because they possess better blast resistance than sandwich structures with monolithic cores [12,13,14]. A graded foam core in a sandwich structure shows great potential to be effective for structural design to improve the overall impact/blast resistance. Graded foam has been receiving increasing attention recently because of its remarkable blast resistance, and its properties can be easily designed and controlled [15,16,17]. Shen et al. [18] found that only one densification wave front appears in cellular materials with a positive gradient, whereas two densification wave fronts appear in cellular materials with a negative gradient. Liu et al. [19] developed theoretical solutions for cellular materials with different density distributions, such as linear, quadratic, and square root. Zhang et al. [20] analyzed the dynamic response of layered cellular materials under impulse loading by using the Voronoi material model. Liang et al. [21,22] investigated the blast behaviors of 1D foam materials with different distributions and developed corresponding theoretical models.

Taking into consideration the damages produced by blasts, blast protection devices need to be improved, because traditional blast-resistant devices shows low efficiency and high cost [23]. Composite sandwich structures have been developed for high blast resistance performance because of their potential for foam core absorbing energy and limited force transfer to protected objects compared with equivalent monolithic counterparts [24]. Sandwich-walled tubes have been proposed as a novel container for temporary storage or transportation of explosive substances [25]. However, investigations on the dynamic response of sandwich-walled cylinders under internal blast loading are precious limited due to the complicated loading behavior caused by fluid–structure interaction and multiple reflections of blast pressure [26]. Shen et al. [27] reported the dynamic behaviors of short sandwich-wall cylinders under internal blast loading and found that the composite structures offer better blast resistance compared with traditional tubes. Karagiozova et al. [28] developed a theoretical solution for the deformation of sandwich-walled cylinders to investigate core densification and face-sheet behavior. Recently, Liang et al. [29] conducted internal blast experiments and simulations on sandwich tubes. Results confirmed that the maximum deformation of sandwich cylinders is sensitive to core, internal face-sheet, and charge mass.

Although several investigations on the dynamic behaviors of sandwich cylinders/rings were reported in the literature, no appropriate analytical model for the deformation of sandwich cylinders/rings with graded cores subjected to internal blast loading has been proposed [30]. A more in-depth understanding of the dynamic response of sandwich cylinders/rings together with the influence of core gradient would assist designers in using graded foam materials in sandwich structures. This paper presents the results of an investigation on the blast response of metal sandwich cylinders/rings with typical double-layered foam cores under internal blast loading. Theoretical and numerical studies were performed to obtain insight into the factors governing the face-sheet deformation and core crushing. In addition, the Voronoi material model is used to simulate the dynamic behaviors of sandwich-walled cylinders under blast loading. The theoretical predictions are compared with finite element (FE) results, and validation of the analytical model of sandwich-walled cylinders is demonstrated. The blast responses of sandwich-walled cylinders are investigated to clarify the effects of core gradient, face-sheet thickness, and charge loading.

## 2. Analytical Model

**Figure 1:**
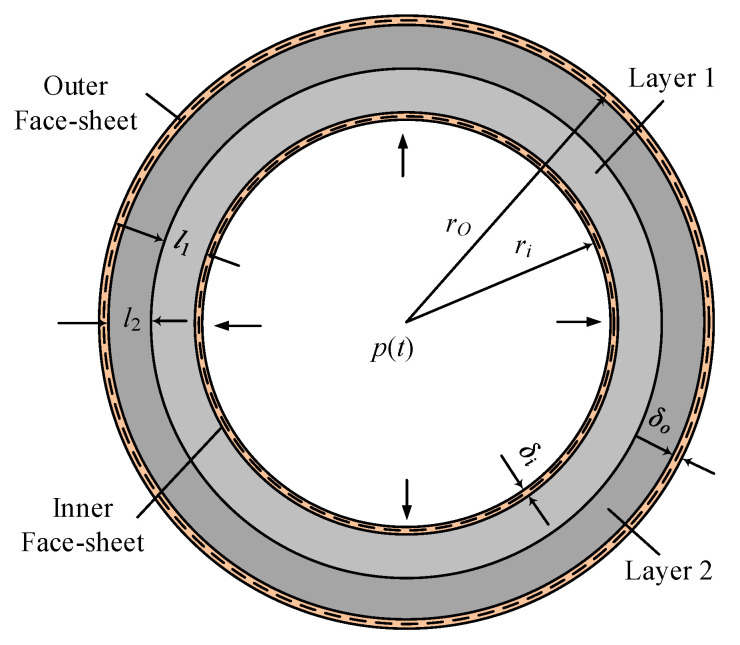
Schematic of the cross-section of a sandwich cylinder/ring.

A theoretical analysis is conducted to provide an estimate of the dynamic response and EA of sandwich cylinders/rings under internal air blast. Such an estimate is expected to provide further insights into the design of graded foam-cored sandwich-walled rings with better blast resistance than monolithic foam-cored rings. Typically, in double-layered cores, the gradient is positive when the soft core is placed inside [31]. In Figure 1, the structure of the cross-section of a cylinder is presented.

The inner and outer face-sheets are made of metal shells with a thickness of *t_i_* and *t_o_* and radius of *r_i_* and *r_o_*. The face-sheets are assumed a rigid–ideally plastic material with a yield strength of *σ_Y_* and density of *ρ_f_*. The core is made of double-layer foam with a thickness of *l*_1_ and *l*_2_. The densities and corresponding plateau stresses of layers 1 and 2 are *ρ*_1_ and *ρ*_2_, and *σ*_1_ and *σ*_2_, respectively. The core gradient is positive when *ρ*_1_ < *ρ*_2_. Cores with positive and negative gradients (*ρ*_1_ < *ρ*_2_ and *ρ*_1_ > *ρ*_2_) are considered in this study.

Dynamic crushing of the foam core has been proposed in the literature based on 1D “shock wave” theory. Shock wave is considered a shock-like densification wave with a fast-propagating thin crushing layer called wave front, which separates the compacted and undeformed regions. Reid and Peng [32] studied shock wave theory to doctrinaire the densification enhancement of a wood material, and they proposed the simplified rigid–perfectly plastic–locking (R-PP-L) material model. The crushing stress *σ_d_* of the shock front is considered a function of propagation velocity *v* through the conservation of mass and momentum at the shock front and by idealizing a cellular material as the R-PP-L model [33]:

(1) $$\sigma_{d}=\sigma_{0}+\frac{\rho v^{2}}{\varepsilon_{d}}$$

where *σ*_0_ and *ε_d_* are the plateau stress and densification of the foam material, respectively.

The size of the foam cell is smaller than its core thickness. Similar to what has been conducted in previous studies, the foam core is considered as a homogeneous material with a strictly concave stress–strain curve in this study, and the topology of foam materials is disregarded. The R-PP-L material model and the shock wave theory are used to analyze blast resistance situations of foam core crushing. The core displays constant compressive stress in the transverse direction of the face-sheet with no lateral expansion up to a particular densification strain. Neither the axial nor longitudinal tensile strengths of the foam core are regarded. In general, impact loading slightly influences the densification strain of the core [34]. However, the densification strain could be approximately regarded as a constant value because blast loading is extremely high [31]. Face-sheet thickness is also assumed a constant value in the expansion process because its thickness is lower than that of the core.

**Figure 2:**
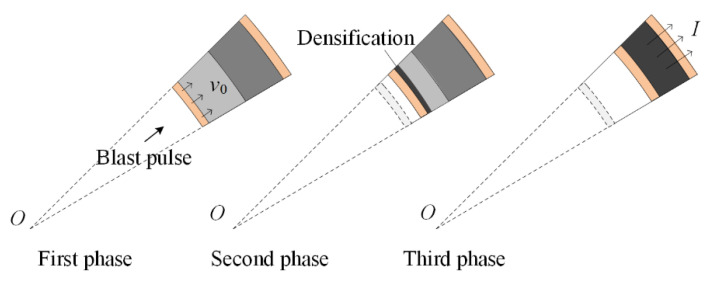
Three response phases for the blast response of a sandwich-walled cylinder.

The dynamic response of face-sheets and the process of compaction wave that propagates through the foam core are combined to propose analytical solutions. The blast response of a sandwich-walled ring is modeled as a three-phase analysis frame (Figure 2). First, in the fluid–structure interaction (FSI) phase, the blast impulse accelerates the inner face-sheet to an initial velocity of *v*_0_. In the core-crushing phase, the inner face-sheet with the initial velocity impacts the core. In the outer face-sheet deformation phase, the outer face-sheet begins to deform after the core compacts fully.

### 2.1. First Phase: FSI

The actual loading on this face-sheet due to an air blast is the reflected over-pressure because FSI affects the air blast and the inner face-sheet. The over-pressure of this incident blast wave, *p_i_*(*t*), impinges on the structure, leading to a reflected over-pressure *p*(*t*). The pressure of the air blast decays exponentially. Generally, it can be approximated as an equivalent triangular pressure pulse, and the pressure could be expressed as

(2) $$p\left(t\right)=\{\begin{array}{cc} p_{0}\left(1-t/t_{0}\right) & t\leq \;\tau_{0}\\ 0 & t>\tau_{0} \end{array}$$

where *p*_0_ is the peak reflected over-pressure of the air blast loading, *τ*_0_ is the air blast loading duration, and *t* is the time.

The initial velocity of the inner face-sheet can be obtained on the basis of the conservation of momentum as the following:

(3) $$v_{0}=\frac{\int_{0}^{\tau_{0}}p\left(t\right)dt}{\rho_{f}\delta_{i}}$$

where *p*(*t*) is the pressure history of the loading, and *ρ_f_* and *δ_i_* are the density and thickness of the inner face-sheet, respectively. The initial velocity of core crushing is obtained by ignoring the slight crushing process of the core in the first phase and combining Equations (2) and (3) as follows:

(4) $$v_{0}=\frac{p_{0}\tau_{0}}{2\rho_{f}\delta_{i}}\;\;t>\tau_{0}$$

### 2.2. Second Phase: Core Crushing

The governing equations of the positive- and negative-gradient double-layer foam core-crushing processes of the foam-cored ring are deduced. Different from the dynamic behavior of the homogeneous foam-cored sandwich-walled ring, the deformation process of the double-layer foam-cored sandwich-walled ring is related to the core gradient.

#### 2.2.1. Positive Gradient Core Situation

**Figure 3:**
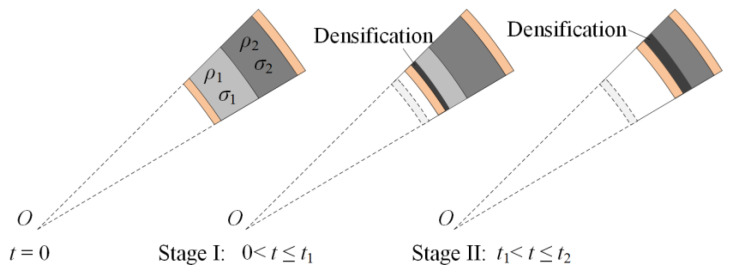
Response process of a cylinder with a positive-gradient core.

For a positive foam core, a low-density layer is placed inside (*ρ*_1_ < *ρ*_2_ and *σ*_1_ < *σ*_2_). The reaction stress at the interface between two layers is the plateau stress of the low-density foam core, *σ*_1_. As the reaction stress is still below the plateau stress of layer 2, the hard layer remains undeformed during the time before layer 1 is completely crushed. Consequently, a densification wave initially occurs in layer 1. Then, the compaction wave propagates to the outer layer after layer 1 compacts completely. As shown in Figure 3, the response process is divided into two stages when the core is a positive gradient.

**Figure 4:**
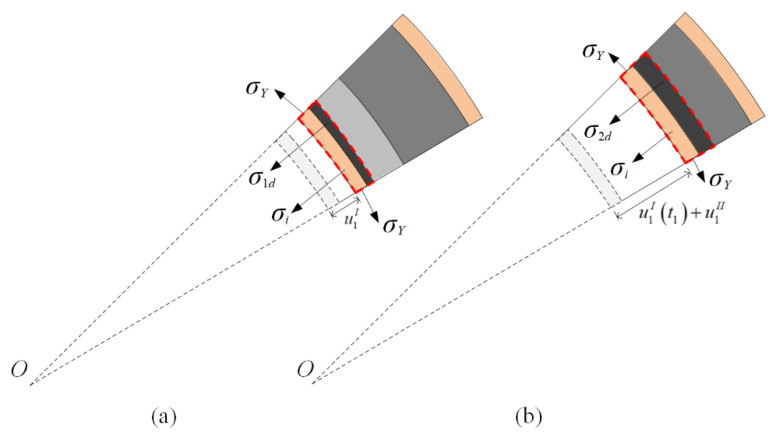
Stress analysis for a cylinder with a positive-gradient core. (**a**) Stage I, and (**b**) stage II.

In stage I, the compaction wave prorogates until the front of the crushing wave arrives at the interface. The displacements of the inner faces of the two layers in stages I and II are $u_{1}^{I}$ and $u_{2}^{I}$ and $u_{1}^{II}$ and $u_{2}^{II}$, respectively. The velocities of the inner faces of the two layers in stages I and II are $v_{1}^{I}$ and $v_{2}^{I}$ and $v_{1}^{II}$ and $v_{2}^{II}$, respectively. The superscript and subscript refer to stage and layer numbers, respectively. Face-sheets are perfectly rigid–plastic, and maintain a constant flow stress value of *σ_Y_*. Figure 4 shows that the circumferential stress causes radial stress in the inner face-sheet as follows:

(5) $$\sigma_{i}=\sigma_{Y}t_{i}/\left(r_{i}+u_{1}^{I}\right)$$

where *r_i_* is the radius of the inner face-sheet.

Figure 4a shows that the velocity of the crushed part is equal to the inner face-sheet in stage I. The motion of the inner face-sheet is given as

(6) $$\sigma_{1d}+\sigma_{i}=-\left[\rho_{f}t_{i}+\left(\rho_{1}/\varepsilon_{1d}\right)u_{1}^{I}\right]\frac{dv_{1}^{I}}{dt}$$

where *σ*_1*d*_ is the crushing stress on the wave front in layer 1, and *ε*_1*d*_ is the densification strain of layer 1. By combining Equations (1), (5), and (6), the velocity of the inner face-sheet and compacted part in stage I, $v_{1}^{I}$, is given as

(7) $$\frac{dv_{1}^{I}}{dt}=\frac{-\sigma_{1}-\left(\rho_{1}/\varepsilon_{1d}\right){\left(v_{1}^{I}\right)}^{2}-\sigma_{Y}t_{i}/\left(r_{i}+u_{1}^{I}\right)}{\rho_{f}t_{i}+\left(\rho_{1}/\varepsilon_{1d}\right)u_{1}^{I}}$$

(8) $$\frac{du_{1}^{I}}{dt}=v_{i}^{I}$$

where *ρ*_1_ is the foam density of layer 1.

The initial conditions are

(9) $$v_{1}^{I}\left(0\right)=v_{0}\begin{array}{cc} , & u_{1}^{I}\left(0\right) \end{array}=0$$

Assume that layer 1 does not deform further in stage II. The compaction part and inner face-sheet are considered rigid bodies to compress layer 2. In Figure 4b, the equations of motion in stage II are given as follows:

(10) $$\frac{dv_{1}^{II}}{dt}=\frac{dv_{2}^{II}}{dt}=\frac{-\sigma_{2}-\left(\rho_{2}/\varepsilon_{2d}\right){\left(v_{1}^{II}\right)}^{2}-\sigma_{Y}t_{i}/\left(r_{i}+u_{1}^{I}\left(t_{1}\right)+u_{1}^{II}\right)}{\left(\rho_{f}t_{i}+\rho_{1}l_{1}\right)+\left(\rho_{2}/\varepsilon_{2d}\right)u_{1}^{II}}$$

(11) $$\frac{du_{1}^{II}}{dt}=v_{1}^{II}$$

(12) $$u_{1}^{I}\left(t_{1}\right)=l_{1}\left(1-\varepsilon_{1d}\right)$$

where *σ*_2*d*_ is the crushing stress on the wave front in layer 2, and *ε*_2*d*_ is the densification strain of layer 2. Moreover, the initial conditions could be given as

(13) $$u_{1}^{II}\left(t_{1}\right)=0\begin{array}{cc} & v_{1}^{II}\left(t_{1}\right)= \end{array}v_{1}^{I}\left(t_{1}\right)$$

#### 2.2.2. Negative-Gradient Core Situation

**Figure 5:**
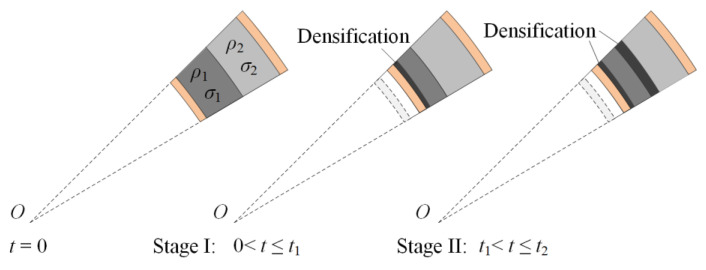
Response process of the core with a negative gradient.

For a negative core, the low-density layer is placed outside (*ρ*_1_ > *ρ*_2_ and *σ*_1_ > *σ*_2_). The reaction stress at the interface is equal to the plateau stress of the inner layer, *σ*_1_, which exceeds the plateau stress of the soft layer, *σ*_2_. Young’s modulus approaches infinity when the core is regarded as the R-PP-L material model. Therefore, the critical velocity of the foam material is approximate to zero. Thus, the face near the blast end crushes first. Subsequently, a new densification wave begins at layer 2 when the stress wave reaches the interface between the two layers. Two compaction waves propagate from the two inner faces of the two layers to their outer faces in the same direction. As shown in Figure 5, the crushing process could be divided into two stages.

In stage I, double compaction waves simultaneously occur in layers 1 and 2 in the same direction when 0 < *t* ≤ *t*_1_. The densification part masses $m_{s1}^{I}$ and $m_{s2}^{I}$ in stage I are derived as

(14) $$m_{s1}^{I}=\rho_{1}\left(u_{1}^{I}-u_{2}^{I}\right)/\varepsilon_{1d}$$

(15) $$m_{s2}^{I}=\rho_{2}u_{2}^{I}/\varepsilon_{2d}$$

where *ρ*_2_ is the foam density of layer 2.

**Figure 6:**
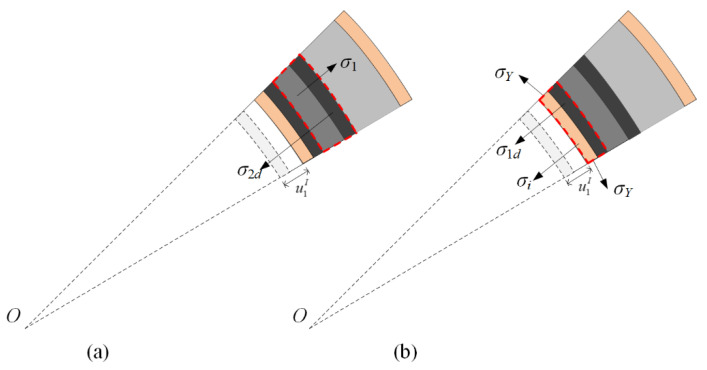
Stress analysis for a cylinder with a negative-gradient core in stage I. (**a**) The undeformed part of layer 1 and the compaction part of layer 2, and (**b**) the compacted part of layer 1.

As shown in Figure 6a, the momentum conservation of the undeformed part of layer 1 and the compaction part of layer 2 gives

(16) $$\sigma_{1}-\sigma_{2d}=\left[\rho_{1}l_{1}-m_{s1}^{I}+m_{s2}^{I}\right]\frac{dv_{2}^{I}}{dt}$$

where $dv_{2}^{I}$ is the compaction wave velocity of layer 2 in stage I.

Substituting Equations (1), (14), and (15) into Equation (16) yields

(17) $$\frac{dv_{2}^{I}}{dt}=\frac{\sigma_{1}-\sigma_{2}-\left(\rho_{2}/\varepsilon_{2d}\right){\left(v_{2}^{I}\right)}^{2}}{\rho_{1}l_{1}-\rho_{1}\left(u_{1}^{I}-u_{2}^{I}\right)/\varepsilon_{1d}+\rho_{2}u_{2}^{I}/\varepsilon_{2d}}$$

(18) $$\frac{du_{2}^{I}}{dt}=v_{2}^{I}$$

The initial conditions are

(19) $$v_{2}^{I}\left(0\right)=0,\;\;u_{2}^{I}\left(0\right)=0$$

The compaction wave velocity in layer 1 relative to that in layer 2 is $v_{1}^{I}-v_{2}^{I}$. According to the stress analysis in Figure 6b, the velocity of the inner face-sheet relative to that of the compacted part in stage I, $v_{1}^{I}-v_{2}^{I}$, is given as the motion equation as follows:

(20) $$\frac{d\left(v_{1}^{I}-v_{2}^{I}\right)}{dt}=\frac{-\sigma_{1}-\left(\rho_{1}/\varepsilon_{1d}\right){\left(v_{1}^{I}-v_{2}^{I}\right)}^{2}-\sigma_{Y}\delta_{i}/\left(r_{i}+u_{1}^{I}\right)}{\rho_{f}\delta_{i}+\rho_{1}\left(u_{1}^{I}-u_{2}^{I}\right)/\varepsilon_{1d}}$$

(21) $$\frac{d\left(u_{1}^{I}-u_{2}^{I}\right)}{dt}=v_{1}^{I}-v_{2}^{I}$$

The initial conditions are

(22) $$v_{1}^{I}\left(0\right)=0,\;u_{1}^{I}\left(0\right)=0,\;v_{2}^{I}\left(0\right)=0,\;u_{2}^{I}\left(0\right)=0$$

Stage II starts at *t* = *t*_1_ when either layer entirely compacts, and finishes at *t* = *t*_2_ when the other layer complete crushes. It is indicated that this stage exists in two scenarios.

Scenario 1: Layer 1 is fully compacted earlier than layer 2. The compaction part mass of layer 2, $m_{s2}^{I}\left(t\right)$, in stage I is derived as

(23) $$m_{s2}^{I}\left(t_{1}\right)=\rho_{2}u_{2}^{I}\left(t_{1}\right)/\varepsilon_{2d}$$

**Figure 7:**
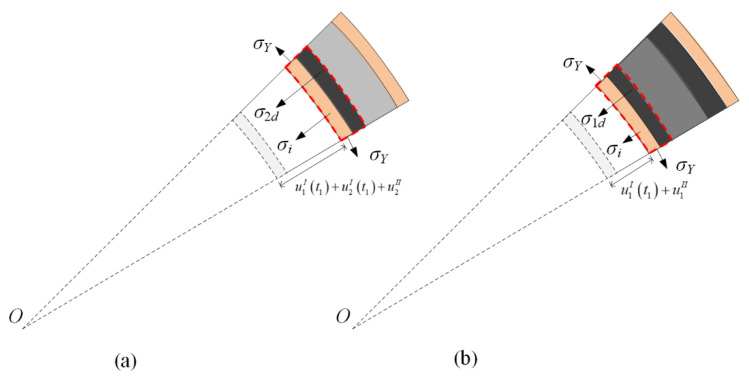
Stress analysis for a cylinder with a negative-gradient core in stage II. (**a**) Scenario 1, and (**b**) Scenario 2.

Figure 7a shows that the conservation of momentum with respect to the inner face-sheet, the compaction part of layer 1, and the compaction part of layer 2 leads to

(24) $$\frac{dv_{2}^{II}}{dt}=\frac{dv_{1}^{II}}{dt}=\frac{-\sigma_{2}-\left(\rho_{2}/\varepsilon_{2d}\right){\left(v_{p}^{II}\right)}^{2}-\sigma_{Y}\delta_{i}/\left(r_{i}+u_{1}^{I}\left(t_{1}\right)+u_{2}^{I}\left(t_{1}\right)+u_{2}^{II}\right)}{\left(\rho_{f}\delta_{i}+l_{1}\rho_{1}+\rho_{2}u_{2}^{I}\left(t_{1}\right)/\varepsilon_{2d}\right)+\left(\rho_{2}/\varepsilon_{2d}\right)u_{2}^{II}}$$

(25) $$\frac{du_{2}^{II}}{dt}=v_{2}^{II}$$

The initial conditions are

(26) $$v_{2}^{II}\left(t_{1}\right)=v_{1}^{II}\left(t_{1}\right)=v_{1}^{I}\left(t_{1}\right),\;\;u_{2}^{II}\left(t_{1}\right)=u_{1}^{II}\left(t_{1}\right)=0$$

Scenario 2: Layer 2 absolutely densifies firstly. The densification part mass of layer 1, $m_{s1}^{I}\left(t\right)$, in stage I is derived as

(27) $$m_{s1}^{I}\left(t_{1}\right)=\rho_{1}u_{1}^{I}\left(t_{1}\right)/\varepsilon_{1d}$$

Figure 7b shows that the momentum conservation of the inner face-sheet and the compacted part of layer 1 leads to

(28) $$\frac{dv_{1}^{II}}{dt}=\frac{-\sigma_{1}-\left(\rho_{1}/\varepsilon_{1d}\right){\left(v_{1}^{II}\right)}^{2}-\sigma_{Y}\delta_{i}/\left(r_{i}+u_{1}^{I}\left(t_{1}\right)+u_{1}^{II}\right)}{\rho_{f}\delta_{i}+\rho_{1}u_{1}^{I}\left(t_{1}\right)/\varepsilon_{1d}+\left(\rho_{1}/\varepsilon_{1d}\right)u_{1}^{II}}$$

(29) $$\frac{du_{1}^{II}}{dt}=v_{1}^{II}$$

The initial conditions are

(30) $$v_{1}^{II}\left(t_{1}\right)=v_{1}^{I}\left(t_{1}\right),\;\;u_{1}^{II}\left(t_{1}\right)=0$$

### 2.3. Third Phase: Outer Face-Sheet Deformation

**Figure 8:**
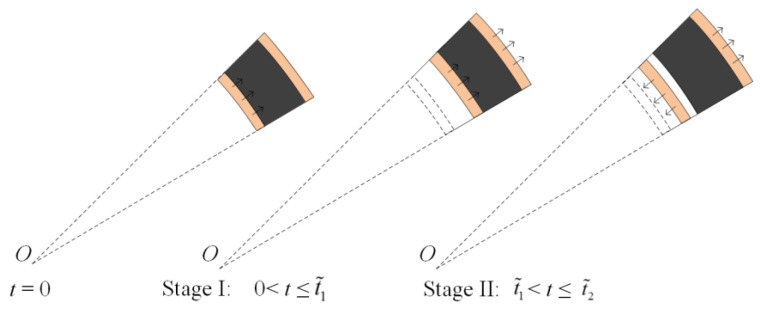
Deformation process of the third phase.

As the core compacts fully, the outer face-sheet starts to deform. Figure 8 shows that the deformation process can be divided into two stages. The velocity of the outer face-sheet initially increases due to the compression of the inner face-sheet and the core. Then, the inner face-sheet separates from the core when the velocities of the inner and outer face-sheets are equal. The velocity of the outer face-sheet begins to decrease.

**Figure 9:**
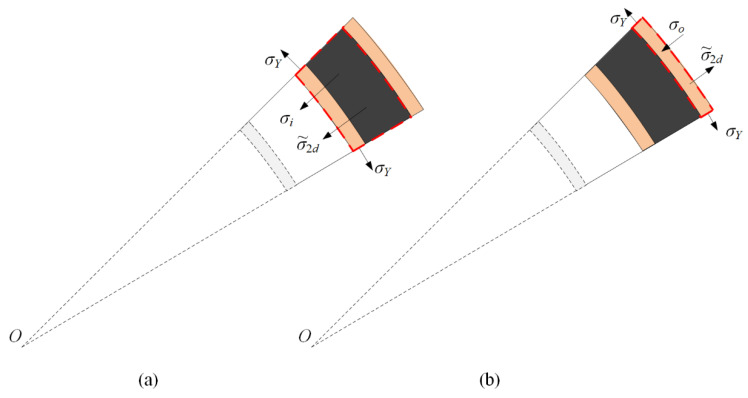
Stress analysis for the sandwich-walled ring in stage I. (**a**) The inner face-sheet and the core, and (**b**) the outer face-sheet.

In stage I, the core, together with the inner face-sheet, compresses the outer face-sheet. Figure 9a shows the stress analysis for the inner face-sheet and the core. The equation of motion for the inner face-sheet is given as follows:

(31) $$\frac{d{\tilde{v}}_{i}^{I}}{dt}=\frac{-\sigma_{Y}t_{i}/\left(r_{i}+l_{1}\varepsilon_{1d}+l_{2}\varepsilon_{2d}+{\tilde{u}}_{i}^{I}\right)-{\tilde{\sigma }}_{2d}}{\rho_{f}t_{i}+\rho_{1}l_{1}+\rho_{2}l_{2}}$$

(32) $$\frac{d{\tilde{u}}_{i}^{I}}{dt}={\tilde{v}}_{i}^{I}$$

where ${\tilde{\sigma }}_{2d}$ is the stress between the core and outer face-sheet. The velocities of the inner face-sheet in stages I and II are ${\tilde{v}}_{i}^{I}$ and ${\tilde{v}}_{i}^{II}$, respectively. The displacements of the inner face-sheet in stages I and II are ${\tilde{u}}_{i}^{I}$ and ${\tilde{u}}_{i}^{II}$, respectively.

The initial conditions are

(33) $${\tilde{\sigma }}_{2d}\left(0\right)=\sigma_{2}+\left(\rho_{2}/\varepsilon_{2d}\right){\left[v_{2}^{II}\left(t_{2}\right)\right]}^{2}$$

(34) $$\begin{array}{cc} {\tilde{u}}_{i}^{I}\left(0\right)=0, & {\tilde{v}}_{i}^{I}\left(0\right)=v_{2}^{II}\left(t_{2}\right) \end{array}$$

Figure 9b shows the stress analysis for the outer face-sheet. The equations of motion for the outer face-sheet are

(35) $$\frac{d{\tilde{v}}_{o}^{I}}{dt}=\frac{-\sigma_{Y}t_{o}/\left(r_{o}+{\tilde{u}}_{o}^{I}\right)+{\tilde{\sigma }}_{2d}}{\rho_{f}t_{o}}$$

(36) $$\frac{d{\tilde{u}}_{o}^{I}}{dt}={\tilde{v}}_{o}^{I}$$

where the velocities of the inner face-sheet in stages I and II are ${\tilde{v}}_{o}^{I}$ and ${\tilde{v}}_{o}^{II}$, respectively. The displacements of the outer face-sheet in stages I and II are ${\tilde{u}}_{o}^{I}$ and ${\tilde{u}}_{o}^{II}$, respectively.

The initial conditions are

(37) $$\begin{array}{cc} {\tilde{u}}_{o}^{I}\left(0\right)=0, & {\tilde{v}}_{o}^{I}\left(0\right)=0 \end{array}$$

**Figure 10:**
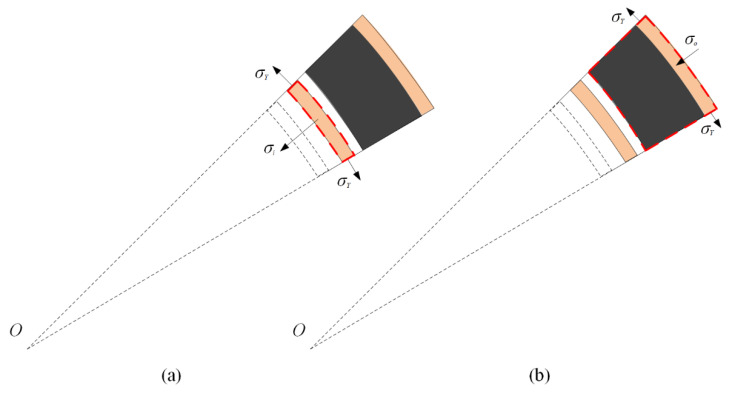
Stress analysis for the sandwich-walled ring in stage II. (**a**) The inner face-sheet, and (**b**) the outer face-sheet.

In stage II, the velocity of the outer face-sheet reaches the maximum and then begins to decrease. The inner face-sheet separates from the core, and the outer face-sheet and the core move together. Figure 10a depicts the stress analysis for the inner face-sheet in stage II. The equations of motion for the inner face-sheet are given as follows:

(38) $$\frac{d{\tilde{v}}_{i}^{II}}{dt}=\frac{-\sigma_{Y}t_{i}/\left(r_{i}+u+{\tilde{u}}_{i}^{I}\left({\tilde{t}}_{1}\right)+{\tilde{u}}_{i}^{II}\right)}{\rho_{f}t_{i}}$$

(39) $$\frac{d{\tilde{u}}_{i}^{II}}{dt}={\tilde{v}}_{i}^{II}$$

The initial conditions are

(40) $$\begin{array}{cc} {\tilde{u}}_{i}^{II}\left(0\right)=0, & {\tilde{v}}_{i}^{II}\left(0\right)={\tilde{v}}_{i}^{I}\left({\tilde{t}}_{1}\right)={\tilde{v}}_{o}^{I}\left({\tilde{t}}_{1}\right) \end{array}$$

Figure 10b shows the stress analysis for the outer face-sheet in stage II. The equations of motion for the outer face-sheet are

(41) $$\frac{d{\tilde{v}}_{o}^{II}}{dt}=\frac{-\sigma_{Y}t_{o}/\left(r_{o}+{\tilde{u}}_{o}^{I}\left({\tilde{t}}_{1}\right)+{\tilde{u}}_{o}^{II}\right)}{\rho_{f}t_{o}+\rho_{1}l_{1}+\rho_{2}l_{2}}$$

(42) $$\frac{d{\tilde{u}}_{o}^{II}}{dt}={\tilde{v}}_{o}^{II}$$

The initial conditions are

(43) $$\begin{array}{cc} {\tilde{u}}_{o}^{II}\left(0\right)=0, & {\tilde{v}}_{o}^{II}\left(0\right)={\tilde{v}}_{o}^{I}\left({\tilde{t}}_{1}\right)={\tilde{v}}_{i}^{I}\left({\tilde{t}}_{1}\right) \end{array}$$

## 3. FE Model

### 3.1. Foam Core Modeling

**Figure 11:**
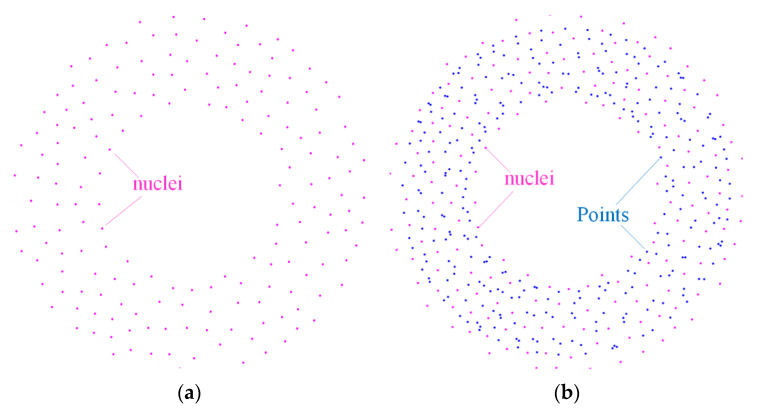

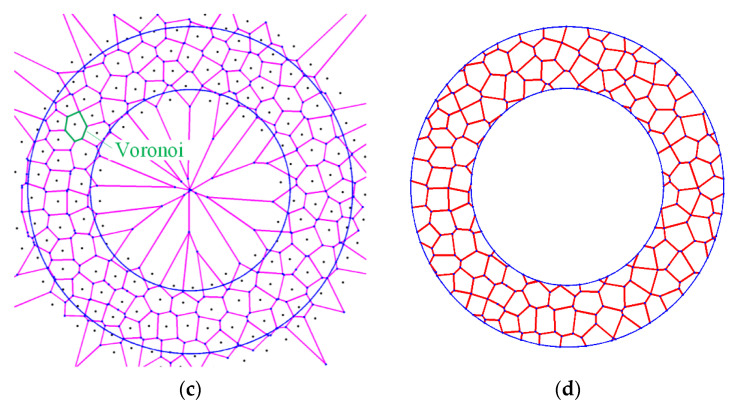
Generated nuclei and constructed Voronoi structure. (**a**) nuclei in given spaces; (**b**) Delaunaytriangulation and Voronoi diagram; (**c**) Voronoi structure. (**d**) foam core achieved.

An analytical model cannot account for natural variations in microstructure that are typical in most foam materials. FE simulations are conducted to verify and provide a detailed description of the blast response of foam-cored sandwich-wall rings. The Voronoi technique is a smart tool used to simulate the deformation process and dynamic behavior of foam materials and sandwich structures. In this study, the foam core is generated using the Voronoi algorithm by the MATLAB 2015a software. Numerical simulation is performed using the ABAQUS/Explicit software. The cells nucleate simultaneously in a given area *A* and grow at an isotropic rate. The irregular degree of foam cell is given as follows:

(44) $$k=1-\delta_{\min}/\delta_{0}$$

where *δ*_min_ and *δ*_0_ are the minimum distance between any two nuclei and between adjacent nuclei, respectively. The process of cell generation can be divided into four stages (Figure 11). First, *N* nuclei, which are constrained to be larger than *δ_min_*, are randomly generated in a given area *A*. The distance between adjacent nuclei is given as

(45) $$\delta_{0}=\sqrt{2A/\sqrt{3}N}$$

Second, points are generated by copying the nuclei to the surrounding region. Then, points close to the nucleus are interconnected, and Delaunay triangulation and Voronoi diagrams are generated. Finally, the Voronoi part is achieved when the area out of the area *A* is deleted. The top and bottom edges in the normal direction of crushing direction were free. The displacement boundary was mainly used in problems related to plastic deformation. The corresponding nodes on the opposite edge of the mesh have the same expansion in the normal direction. Self-contact was defined for all the cell surfaces.

### 3.2. Material Model

The detonation products of charges are described by the Jones–Wilkins–Lee (JWL) model. In the model, the pressure distribution is relative with relative volume and internal energy:

(46) $$p=A\left(1-\frac{\omega V}{R_{1}}\right)e^{-R_{1}V}+B\left(1-\frac{\omega }{R_{2}V}\right)e^{-R_{2}V}+\frac{\omega }{V}E_{0}$$

where *p* is the product pressure, *V* is the relative volume, *A*, *B*, *R*_1_, *R*_2_, and *ω* are charge constants; and *E*_0_ is the initial internal energy. The JWL constants of the charge are listed in Table 1. The FE model of the core is constructed by the Voronoi technique. The base material is aluminum. The cell wall of the core is assumed an elastic–perfectly plastic model. The base material parameters are listed in Table 2.

### 3.3. Numerical Model

**Figure 12:**
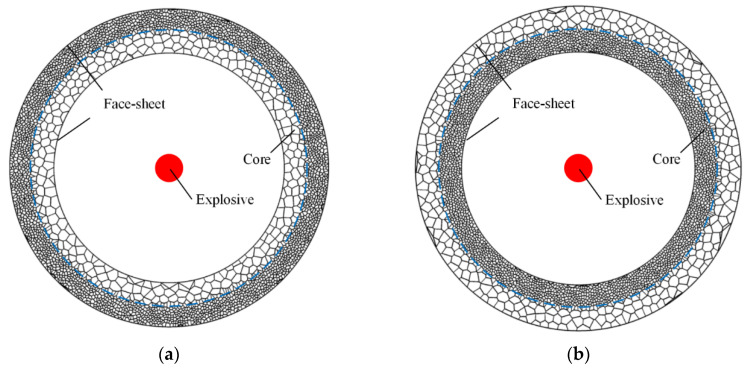
Finite element (FE) models [29]. (**a**) Positive gradient core, and (**b**) negative gradient core.

Numerical simulations are performed with the ABAQUS 6.9/Explicit software. Two typical FE models of sandwich-walled rings are shown in Figure 12. A double-layer core with a positive or negative gradient is sandwiched between face-sheets. The core and face-sheets are modeled using the S4R shell element type. Self-contact is also specified between the face-sheets that may contact other cell faces during crushing. General contact is used for parts. The friction coefficient between the core and face-sheets is 0.02 [36]. A good agreement was achieved between the results of the FE model and corresponding experiments [29].

## 4. Comparisons of Theoretical and Numerical Results

**Figure 13:**
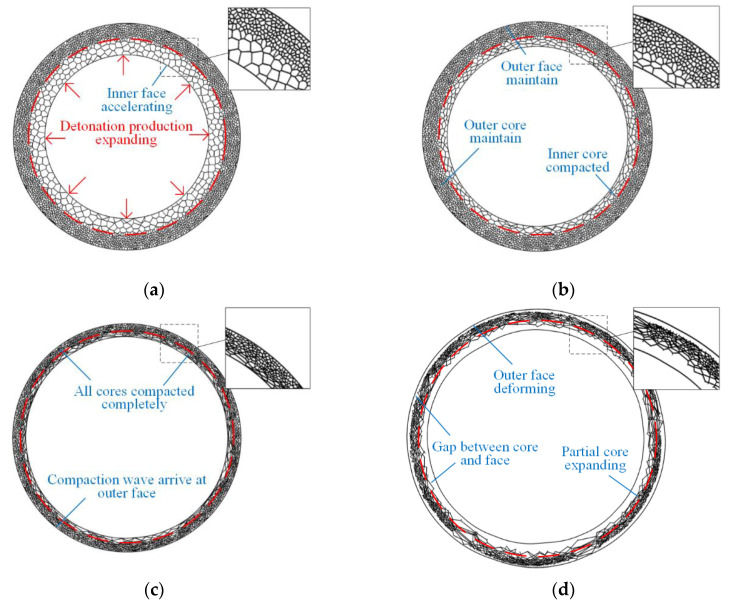
FE results of sandwich rings with positive gradient core. (**a**) First phase, (**b**) second phase compaction wave, (**c**) second phase outer core compacted, and (**d**) third phase.

**Figure 14:**
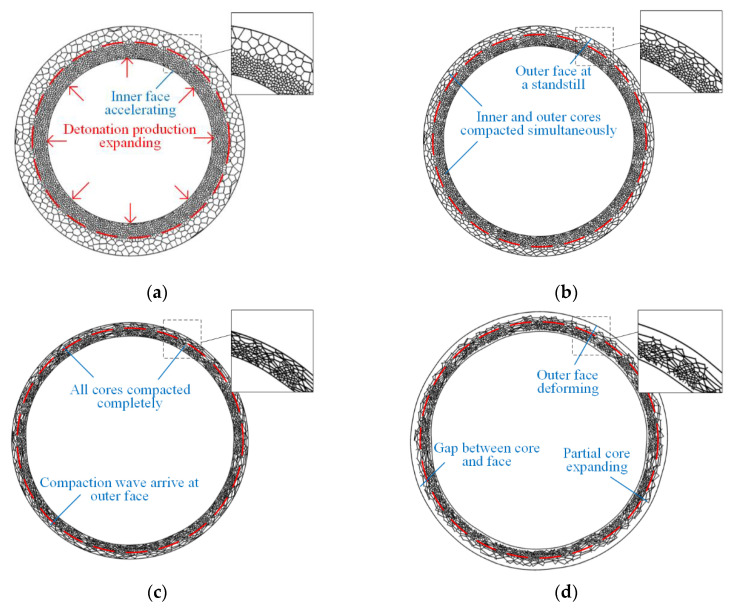
FE results of sandwich rings with negative gradient core. (**a**) First phase, (**b**) second phase, (**c**) end of second phase, and (**d**) third phase.

Comparisons of the FE results for the deformation of the maximum radial deflection (MRD) and analytical predictions based on axisymmetric deformation of a sandwich ring are performed to verify the theoretical solutions. The deformation processes of rings with positive and negative cores are presented in Figure 13 and Figure 14. When the gradient is positive, only one shock wave propagates from the inside to the outside during the complete crushing process. The phenomenon coincides with the analytical prediction. When the gradient is negative, double shock fronts propagate outside in the same direction. However, this phenomenon is different from the continuous-density foam that has double shock fronts with opposite directions [37].

**Figure 15:**
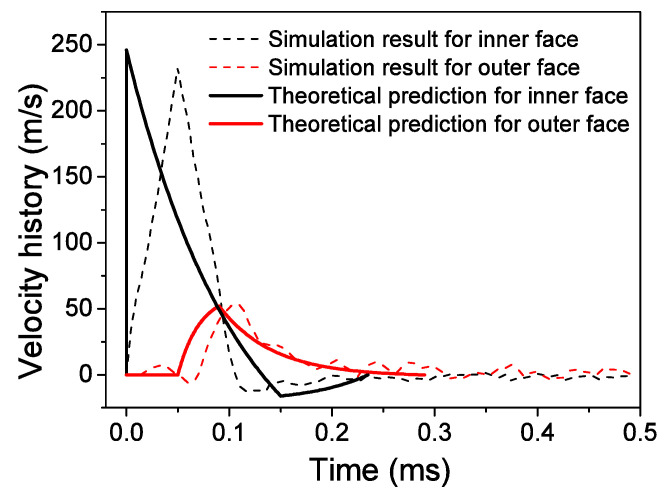
Comparison of face-sheet velocities between theoretical predictions and FE results.

**Figure 16:**
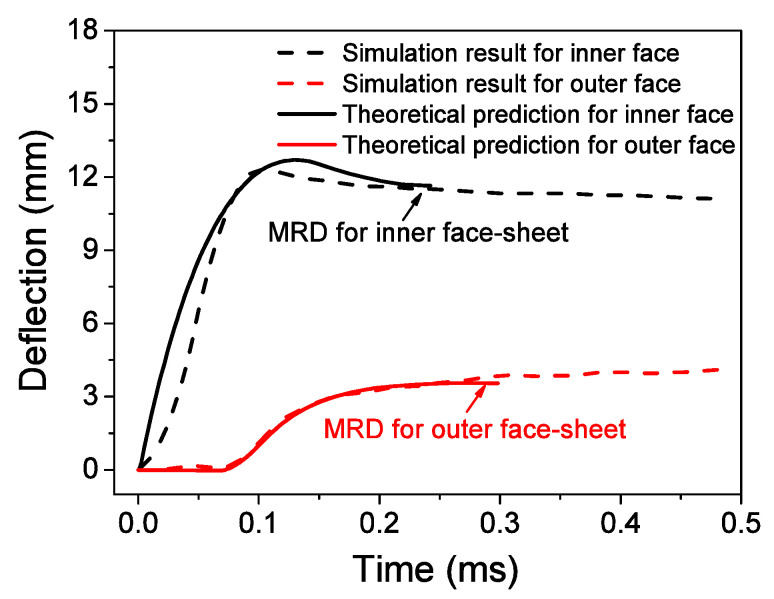
Comparison of maximum radial deflections (MRDs) of face-sheets between theoretical predictions and FE results.

The velocity histories of face-sheets are shown in Figure 15. The theoretical solution results in a larger MRD than the FE results. The theoretical predictions can generally describe the essential features of the three phases obtained by the numerical results. A difference is observed between the numerical simulation and theoretical prediction for the inner face-sheet during the first phase because the interaction between the inner face-sheet and core is neglected in this phase. As shown in Figure 16, reasonable agreement is observed between the theoretical predictions and simulation results for the face-sheets. The theoretical predictions can give a good prediction for the FE results, however, the sudden change between phases in the theoretical model does not coincide with the gradual transitions in the FE results. This deviation is also related to the R-PP-L model used in the theoretical model. The reflected over-pressure history at the location where a possible maximum reflected over-pressure history *p*(*t*) occurs can be measured because the air blast does not have a uniform distribution along the axial direction of the ring. The theoretical results are obtained by using the maximum reflected over-pressure history *p*(*t*). Using theoretical results as a criterion for the design of a structure with limited MRD is conservative.

## 5. Discussion

Determining the plastic dissipation of the core and the MRD of face-sheets for sandwich-walled rings is of practical interest. The sandwich-walled ring with high EA and low MRD is a good choice to maximize the blast resistance of the sandwich-walled ring at a given mass subjected to internal blast loading.

Given that the elastic deformation energy of the core is negligible in comparison with the plastic deformation energy, the plastic dissipation of the layered foam under a quasi-static state can be calculated as follows:

(47) $$E_{0}=l_{1}\sigma_{1}\varepsilon_{1d}+l_{2}\sigma_{2}\varepsilon_{2d}$$

The plastic dissipation under blast loading can be obtained as

(48) $$E=\int_{0}^{l_{1}}\frac{1}{2}\left(\sigma_{1}+\sigma_{1d}\right)\varepsilon_{1d}d\xi +\int_{0}^{l_{2}}\frac{1}{2}\left(\sigma_{2}+\sigma_{2d}\right)\varepsilon_{2d}d\xi$$

where *ξ* is the Lagrangian coordinate of the shock front. 

By substituting Equations (1) and (47) into Equation (48), the above equation can be rewritten as

(49) $$E=E_{0}+\frac{1}{2}\left(\int_{0}^{l_{1}}\rho_{1}v{\left(\xi \right)}^{2}d\xi +\int_{0}^{l_{2}}\rho_{2}v{\left(\xi \right)}^{2}d\xi \right)$$

Based on Equations (31) and (38), the MRD of the inner face-sheet is given as

(50) $$D_{i}=l_{1}\varepsilon_{1d}+l_{2}\varepsilon_{2d}+{\tilde{u}}_{i}^{I}\left({\tilde{t}}_{1}\right)+{\tilde{u}}_{i}^{II}\left({\tilde{t}}_{2}\right)$$

Based on Equations (35) and (41), the MRD of the outer face-sheet is given as

(51) $$D_{o}={\tilde{u}}_{o}^{I}\left({\tilde{t}}_{1}\right)+{\tilde{u}}_{o}^{II}\left({\tilde{t}}_{2}\right)$$

**Figure 17:**
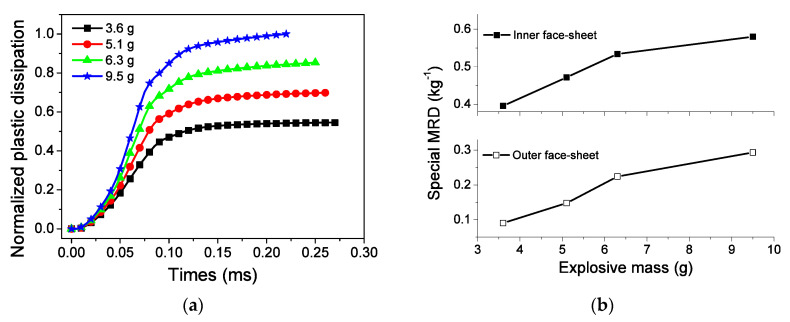
Influence of explosive mass on energy absorption (EA) and MRD. (**a**) EA, and (**b**) MRD.

As shown in Figure 17a, the EA capacity of the sandwich-walled ring with a double-layer core increases with the internal explosive mass. The relationship between EA and explosive mass can be explained as follows. According to Equation (3), a high internal explosive mass leads to a high initial velocity in Phase I, which leads to high crushing stress based on Equation (1). Considering Equation (49), the EA capacity increases with velocity-dependent dynamic enhancement and increasing crushed displacement. Figure 17b shows the effect of explosive mass on the MRDs of the inner and outer face-sheets. The MRDs are small for the sandwich-walled rings under low explosive loading due to the low impulse for face-sheets. This phenomenon coincides with our previous investigation [30]. In addition, the EA of the core increases by 182% as the internal pressure increases 22%. It is indicated that the increase in explosive charge would seriously increase the burden of blast-protection structures, whether in terms of MRD or EA. 

**Figure 18:**
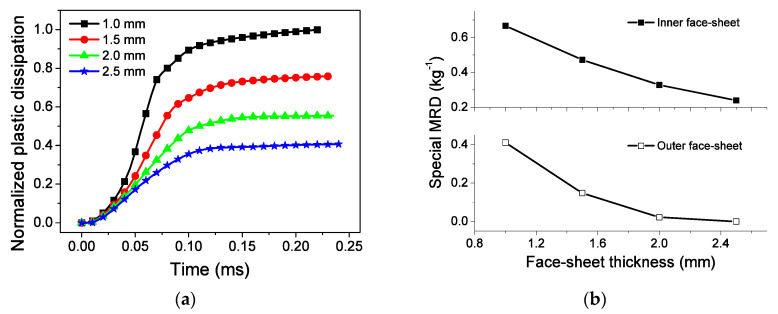
Influence of face-sheet thickness on EA and MRD. (**a**) EA, and (**b**) MRD.

For a given blast loading and core configuration, the sandwich-walled rings with thin face-sheets absorb much energy and deform seriously, as shown in Figure 18. The increase in face-sheet thickness leads to a rise in the mass and stiffness of sandwich-walled rings because of the decrease in energy dissipation. The thin face-sheet can improve the EA capability. However, this phenomenon easily leads to large deformation of the face-sheet, resulting in weak blast resistance. Different from Ref. [29], the thickness of face-sheets varies in a certain range. This leads to a significant increase in blast resistance performance, but the total mass of the structure increases.

**Figure 19:**
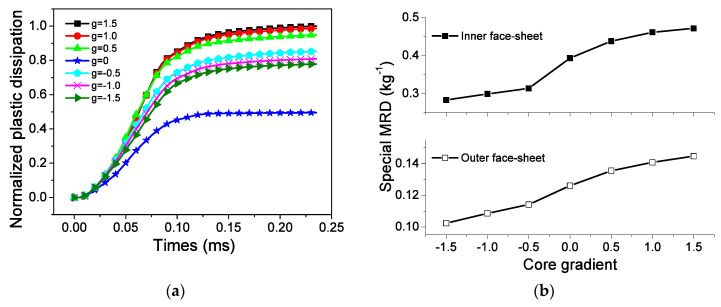
Influence of core gradient on EA and MRD. (**a**) EA, and (**b**) MRD.

The core gradient is defined as

(52) $$g=\frac{\sigma_{2}r_{2}-\sigma_{1}r_{1}}{\left(\sigma_{1}+\sigma_{2}\right)\left(r_{1}+r_{2}\right)}$$

where *r*_1_ and *r*_2_ are the thicknesses of layers 1 and 2, respectively. The core gradient is positive when the low-density layer is located inside. Figure 19 depicts the gradient influence on the EA and MRD of the sandwich-walled ring. For a given geometrical sandwich-walled ring subjected to identical blast loading, the dissipated energy increases as the core gradient increases. The ring with a graded core displays better EA capability compared with the ring with a uniform core. However, the decrease in gradient is attributed to the reduction in the MRDs of face-sheets Figure 19b. EA and MRD are two conflicting objectives to evaluate blast resistance for explosive mass, face-sheet thickness, and core gradient. Karagiozova [28] found that although the core causes a reduction in the maximum velocity, the use of high-density foam is not a way to reduce the displacements. This conclusion is consistent with the phenomenon in Figure 19b.

## 6. Conclusions

Theoretical analysis of the blast protection of graded metal foam-cored sandwich cylinders/rings is performed in this study. The mechanism of deformation is studied for a graded foam-cored sandwich cylinder/ring employing numerical and analytical methods. Several conclusions can be made with respect to the deformation process of graded metal foam-cored sandwich cylinders/rings resulting from an internal blast loading.

The deformation process can be modeled as three phases: FSI phase, core-crushing phase, and outer face-sheet deformation phase. For the core crushing, only one shock wave propagates from the inner layer to the outer layer for a positive-gradient core. Two compaction waves emerging at two internal faces of two layers simultaneously propagate outward in the same direction for a negative-gradient core.

The dynamic responses and EA of sandwich-walled rings with graded foam cores are compared with those of ungraded ones. When graded sandwich-walled rings and ungraded rings are subjected to an identical air blast, the MRD of the former is smaller than that of the latter, whereas the EA of the former is stronger than that of the latter. It is concluded that EA and MRD are two conflicting goals for achieving high impact/blast resistance capability for explosive mass, face-sheet thickness, and core gradient.

## Figures and Tables

**Table 1:** JWL model parameters of the charge [30].

Explosive	*A*	*B*	*ω*	*R*_1_	*R*_2_	*E*_m0_ (J/m^3^)
TNT	3.74	0.032	0.3	4.15	0.95	70

**Table 2:** Base material parameters [35].

Material	Density *ρ_s_* (kg/m^3^)	Young Modulus *E_s_* (GPa)	Poisson Ratio *γ_s_*	Yield Stress *σ_ys_* (MPa)
Aluminum	2730	69.2	0.3	168
